# High-throughput karyotyping of human pluripotent stem cells

**DOI:** 10.1016/j.scr.2012.06.008

**Published:** 2012-11

**Authors:** Riikka J. Lund, Tuomas Nikula, Nelly Rahkonen, Elisa Närvä, Duncan Baker, Neil Harrison, Peter Andrews, Timo Otonkoski, Riitta Lahesmaa

**Affiliations:** a Molecular and Systems Immunology and Stem Cell Biology, Turku Centre for Biotechnology, University of Turku and Åbo Akademi University, FI-20520 Turku, Finland; b PerkinElmer Diagnostics, P.O. Box 10, FI-20101 Turku, Finland; c Centre for Stem Cell Biology, the Department of Biomedical Science, University of Sheffield, Sheffield S10 2TN, UK; d Program of Molecular Neurology, Biomedicum Stem Cell Center, University of Helsinki, FI-00290 Helsinki, Finland; e Children's Hospital, Helsinki University Central Hospital, FIN-00029 Helsinki, Finland

## Abstract

Genomic integrity of human pluripotent stem cell (hPSC) lines requires routine monitoring. We report here that novel karyotyping assay, utilizing bead-bound bacterial artificial chromosome probes, provides a fast and easy tool for detection of chromosomal abnormalities in hPSC lines. The analysis can be performed from low amounts of DNA isolated from whole cell pools with simple data analysis interface. The method enables routine screening of stem cell lines in a cost-efficient high-throughput manner.

## Highlights

► We report a novel high-throughput karyotyping assay for human pluripotent cells. ► This assay provides fast and easy tool for detection of chromosomal abnormalities. ► The analysis can be run from low DNA amounts with simple data analysis interface. ► The results are in good concordance with the data from G-banding and SNP arrays. ► The assay enables routine screening of stem cell lines in a cost-efficient manner.

Primary human embryonic stem cell (hESC) lines, as well as induced pluripotent stem cell (iPSC) lines, provide a valuable resource for developmental studies, disease modeling and development of regenerative therapies. Long term culture of hESC is associated with the accumulation of karyotypic abnormalities (Draper et al., 2004; Cowan et al., 2004; Baker et al., 2007). Based on previous large scale studies on at least 1370 hESC samples, most commonly the recurrent karyotypic abnormalities in hESCs involve partial or whole gains of chromosomes 12, 17, 20 or X (Baker et al., 2007; Laurent et al., 2011; Taapken et al., 2011; Spits et al., 2008; Lefort et al., 2008; The International Stem Cell Initiative et al., 2011). Furthermore, pluripotent reprogramming, required for the derivation of iPSC, is associated with frequent copy number variation (Hussein et al., 2011). Thus, continuous monitoring of the genomic integrity of these lines is important, as genomic alterations may change the developmental potential and malignant capacity of the cells.

Conventional karyotyping has been most commonly used to monitor the genomic status of the cells. The method enables early detection of abnormalities in heterogeneous populations. However, the analysis is usually limited to less than 50 mitosis instead of the whole cell population. Furthermore, conventional karyotyping is costly, slow and laborious hampering the frequency of the analysis. While array based methods enable high resolution analysis of the whole cell population, they are also expensive, slow and require specialized expertise for data analysis and interpretation. Furthermore, the complexity of the array data can be difficult to interpret, as for instance it is not clear which of the small scale aberrations detected in hESCs have been present already in the original blastocyst from which the cells have been derived. For routine and frequent monitoring of the hESC and iPSC karyotypes, fast, simple and inexpensive method would be highly beneficial for laboratories producing, maintaining and utilizing hESCs or iPSCs.

KaryoLite™ BoBs™, developed by PerkinElmer, is a novel improved method for fast and easy molecular karyotyping based on BACs-on-Beads™ technology (Vialard et al., 2011). The assay measures DNA copy numbers at the chromosome arm resolution utilizing bacterial artificial chromosome (BAC) probes immobilized onto color-encoded polystyrene microspheres distinguishable by a Luminex fluorometer. The KaryoLite™ BoBs™ consists of 90 beads, each of which is a composite of three different neighboring BACs based on the available Human Genome Build. The probes are targeted to proximal and terminal regions of each chromosome arm of metacentric and sub-metacentric chromosomes and q-arms of acrocentric chromosomes. As previous studies have indicated that the most common karyotypic abnormalities in hESCs involve gains of whole chromosomes or telomeric regions of chromosomes, it is expected that the frequent and recurrent abnormalities are covered by the probes included in the KaryoLite™ BoBs™ (Baker et al., 2007; Laurent et al., 2011; Taapken et al., 2011).

To compare the novel method to conventional G-banding and array based methods, we reanalyzed seven different hESC lines from our previous detailed genomic analysis (Table 1) by Narva et al. (2010). Most of these samples had been previously analyzed with both G-banding and Affymetrix high resolution SNP6.0 arrays. In addition, new samples of H9 line, with normal (H9p35 and H9p38) or abnormal (H9p99 and H9p112) karyotype were included in the analysis. This panel of samples represents karyotypes ranging from normal to most commonly observed recurrent chromosomal aberrations in hESCs lines (Baker et al., 2007; Laurent et al., 2011; Taapken et al., 2011; Spits et al., 2008; Lefort et al., 2008). Selected sample material was used to examine capacity of KaryoLite™ BoBs™ in the detection of known abnormalities in hESCs and sensitivity to detect the changes in karyotypically heterogeneous cell populations. Altogether 12 samples (7 hESC lines), 7 normal and 6 abnormal, including 2 lines with mosaic karyotypes, were studied with KaryoLite™ BoBs™ assay. From each sample at least 100 ng of DNA was used as a starting material for the karyotypic analysis. The samples were processed according to the instructions described in the KaryoLite™ BoBs™ manual (Product Number 4501–0010, Perkin Elmer, Turku, Finland). According to the assay workflow (Supplementary Fig. 1), the sample DNA is labeled with Biotin, purified and hybridized to beads with complementary BAC probes. After washing and reporter (streptavidin-PE) binding the fluorescent signals are measured with Luminex instrument and results analyzed with BoBsoft™ analysis software (Perkin Elmer). In this study female and male genomic DNA from the karyotypically normal hESC lines were used as a reference. Alternatively, commercial female and male reference DNA can be used. The BoBsoft™ depicts karyotypes as a ratio plots against female and male reference signals of normal karyotype (either measured from external DNA samples or calculated for each bead from the normally behaving beads in the assay data). The user defined threshold values for karyotypic changes are generated by the BoBsoft data analysis software and are multiples of standard deviation, calculated from beads with ratios < 2SD from 1.

To compare the novel method to conventional G-banding and array based methods, we reanalyzed seven different hESC lines from our previous detailed genomic analysis (Table 1) by Narva et al. (2010). Most of these samples had been previously analyzed with both G-banding and Affymetrix high resolution SNP6.0 arrays. In addition, new samples of H9 line, with normal (H9p35 and H9p38) or abnormal (H9p99 and H9p112) karyotype were included in the analysis. This panel of samples represents karyotypes ranging from normal to most commonly observed recurrent chromosomal aberrations in hESCs lines (Baker et al., 2007; Laurent et al., 2011; Taapken et al., 2011; Spits et al., 2008; Lefort et al., 2008). Selected sample material was used to examine capacity of KaryoLite™ BoBs™ in the detection of known abnormalities in hESCs and sensitivity to detect the changes in karyotypically heterogeneous cell populations. Altogether 12 samples (7 hESC lines), 7 normal and 6 abnormal, including 2 lines with mosaic karyotypes, were studied with KaryoLite™ BoBs™ assay. From each sample at least 100 ng of DNA was used as a starting material for the karyotypic analysis. The samples were processed according to the instructions described in the KaryoLite™ BoBs™ manual (Product Number 4501–0010, Perkin Elmer, Turku, Finland). According to the assay workflow (Supplementary Fig. 1), the sample DNA is labeled with Biotin, purified and hybridized to beads with complementary BAC probes. After washing and reporter (streptavidin-PE) binding the fluorescent signals are measured with Luminex instrument and results analyzed with BoBsoft™ analysis software (Perkin Elmer). In this study female and male genomic DNA from the karyotypically normal hESC lines were used as a reference. Alternatively, commercial female and male reference DNA can be used. The BoBsoft™ depicts karyotypes as a ratio plots against female and male reference signals of normal karyotype (either measured from external DNA samples or calculated for each bead from the normally behaving beads in the assay data). The user defined threshold values for karyotypic changes are generated by the BoBsoft data analysis software and are multiples of standard deviation, calculated from beads with ratios < 2SD from 1.

**Figure 1:**
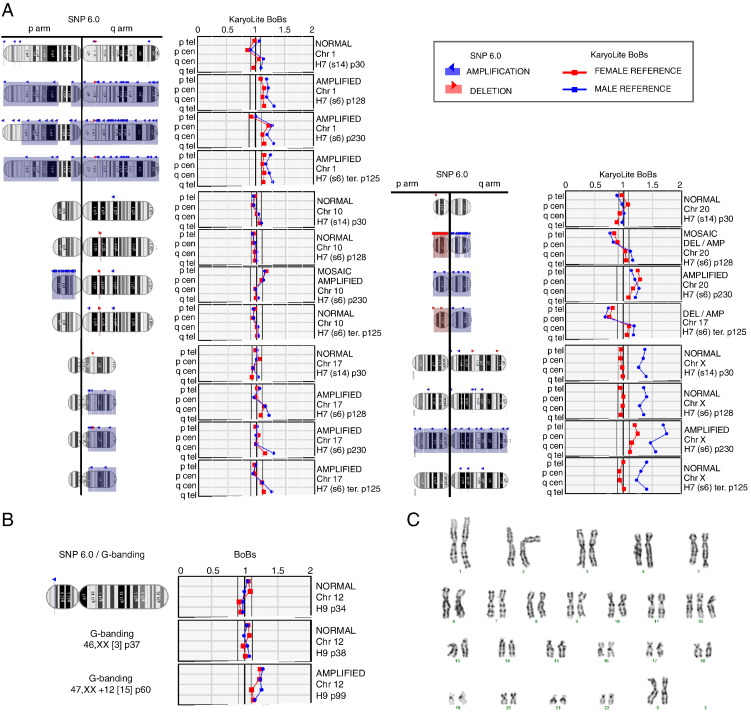
Karyotypic changes detected by KaryoLite™ BoBs™ assay in human embryonic stem cells in comparison to Affymetrix SNP6.0 arrays or G-banding. In Fig. 1A (H7 line) and B (H9 line) are the abnormalities detected by KaryoLite™ BoBs™ assay (right panel) in comparison to Affymetrix SNP6.0 arrays and/or Chromosome G-banding method (left panel). In the left panel the chromosomal areas painted with red color indicate losses detected with Affymetrix SNP6.0 arrays, whereas blue indicates gains. In the right panel the red and blue lines indicate chromosomal signal ratios against female (red) and male (blue) reference DNA with normal karyotypes as detected by KaryoLite™ BoBs™ assay. For the normal chromosomes both the signals calculated against male and female references should reside inside the reference area around value 1, whereas for the abnormal karyotype both signals should be outside the reference area. In Fig. 1C are the representative cytogenetic data for H9 line with karyotype 47,XX,+12.

**Figure 2:**
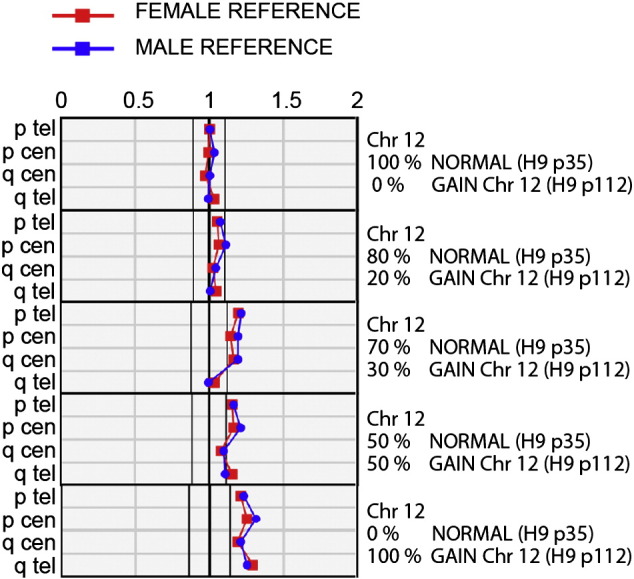
Detection threshold of mosaic trisomy 12. Human H9p35 embryonic stem cells with normal karyotype were pooled in different proportions with H9p112 karyotypically abnormal cells carrying trisomy of chromosome 12 as indicated in the figure. The karyotypes were analyzed with Karyolite BoBs assay to estimate the threshold for detection of mosaic chromosomal abnormalities.

As expected, the analysis results did not indicate any large scale genomic abnormalities in the karyotypically normal hESC lines (H7p30, H9p25, H9p35, H9p38, HS401p53, HS293p60 or FES22p41) (Narva et al., 2010). For the abnormal cell lines (H7p128, H7p230, H7 teratoma p125, H9p99 or H9p112) all the large scale gains detected with karyotyping and or SNP6.0 arrays in the chromosomes 1, 12, 17, 20 or X were detected also with KaryoLite™ BoBs™ assay (Fig. 1). Even the mosaic isochromosome 20(q10) present in 5 of the 20 metaphases (25%) of H7p128 line was detected as a deletion of telomeric area of the p-arm. For the same sample previous SNP6.0 array revealed gain of the p-arm and loss of q-arm. Interestingly, both KaryoLite™ BoBs™ and Affymetrix SNP6.0 arrays detected gain of Chromosome 10 in the H7p230 sample, which was not detected by conventional G-banding method. Similarly to SNP6.0 arrays, none of the balanced translocations detected with G-banding were detected by bead based system. To examine the detection threshold for mosaic karyotypes, H9 cells with trisomy of chromosome 12 were mixed with karyotypically normal cells in 0%, 20%, 30%, 50% and 100% proportions. The results show that when the proportion of karyotypically abnormal cells is at least ≥ 30% within the cell population they can be detected by the KaryoLite™ BoBs™ (Fig. 2). Thus, the mosaicism of cell line FES29p37, previously reported with the karyotype 46,XY, add(13)(p1)[1]/46,XY[30], was not detected by either KaryoLite™ BoBs™ or previously by SNP6.0 arrays. In conclusion, the data obtained with bead based system are in good concordance with the previous analysis performed using G-banding method and Affymetrix SNP6.0 arrays.

These results highlight that the novel KaryoLite™ BoBs™ assay provides a valuable tool for routine screening of the recurrent and random chromosomal abnormalities observed in hESC lines. The method is most likely to be applicable for monitoring of iPSC lines as well, although these were not included in this study. The analysis with KaryoLite™ BoBs™ can be carried out in a 96-well format enabling scaling up the sample numbers. The data analysis interface is simple to use and the results are clear and easy to interpret. The sensitivity of the method may also enable detection of large scale karyotypic aberrations in mosaic populations. The benefit of the method in comparison to conventional karyotyping also includes examination of the whole cell population rather than fraction of the cells (typically < 50 mitosis) providing improved representation of the genomic integrity of the whole cell population (Supplementary Table 1). The method is not suitable for detecting balanced translocations or with current probe selection sensitive enough for small scale changes. However, most of the recurrent genomic changes in pluripotent cells involve whole chromosomes or chromosome arms (Baker et al., 2007; Laurent et al., 2011; Taapken et al., 2011; Spits et al., 2008; Lefort et al., 2008; The International Stem Cell Initiative et al., 2011). Also for detection of low level of mosaicisms the conventional G-banding is more sensitive method. For important samples a thorough analysis utilizing more sensitive methods detecting balanced rearrangement and also small copy number variations is recommended. In conclusion, the novel method described here provides rapid, high-throughput and cost-efficient tool for frequent monitoring and screening of the genomic integrity of stem cell cultures.

These results highlight that the novel KaryoLite™ BoBs™ assay provides a valuable tool for routine screening of the recurrent and random chromosomal abnormalities observed in hESC lines. The method is most likely to be applicable for monitoring of iPSC lines as well, although these were not included in this study. The analysis with KaryoLite™ BoBs™ can be carried out in a 96-well format enabling scaling up the sample numbers. The data analysis interface is simple to use and the results are clear and easy to interpret. The sensitivity of the method may also enable detection of large scale karyotypic aberrations in mosaic populations. The benefit of the method in comparison to conventional karyotyping also includes examination of the whole cell population rather than fraction of the cells (typically < 50 mitosis) providing improved representation of the genomic integrity of the whole cell population (Supplementary Table 1). The method is not suitable for detecting balanced translocations or with current probe selection sensitive enough for small scale changes. However, most of the recurrent genomic changes in pluripotent cells involve whole chromosomes or chromosome arms (Baker et al., 2007; Laurent et al., 2011; Taapken et al., 2011; Spits et al., 2008; Lefort et al., 2008; The International Stem Cell Initiative et al., 2011). Also for detection of low level of mosaicisms the conventional G-banding is more sensitive method. For important samples a thorough analysis utilizing more sensitive methods detecting balanced rearrangement and also small copy number variations is recommended. In conclusion, the novel method described here provides rapid, high-throughput and cost-efficient tool for frequent monitoring and screening of the genomic integrity of stem cell cultures.

The following are the supplementary related to this article.

Supplementary Fig. 1 Workflow of the Karyolite Assay.

Supplementary Table 1 Comparison of the three methods used to detect karyotypic abnormalities in this study.

Supplementary related to this article can be found online at http://dx.doi.org/10.1016/j.scr.2012.06.008.

## Figures and Tables

**Table 1:** Human embryonic stem cell samples used in the KaryoLite™ BoBs™ analysis.

hESC line	Passage (p)	Karyotype (G-banding)	Karyotyped (p)	Laboratory
H7.s14^a^	P30	46,XX[20]	P38	P.W.A.
H7.s6^a^	P128	47,XX,+1,der(6)t(6;17)(q27;q1)[15]		
		/47,XX,+1,der(6)t(6;17)(q27;q1),i(20)(q10)[5]	P132	P.W.A.
H7.s6^a^	P230	49,XXX,+add(1)(p3),der(6)t(6;17)(q27;q1),+20[30]	P237	P.W.A.
H7.s6.ter^a^	P125	47,XX,+add(1)(p1),der(6)t(6;17)(q27;q1),i(20)(q10)[30]	P127	P.W.A.
H9^a^	P25	46,XX[20]	P27	R.L.
H9	P35	46,XX[20]	P36	R.L.
H9	P38	46,XX[3 × 2]	P37	R.L.
H9	P99	47,XX,+12[15]	P60	R.L.
H9	P112	47,XX,+12[15]	P60	R.L.
HS401^a^	P53	46,XY[30]	P53	R.L.
HS293^a^	P60	Not determined		R.L.
FES22^a^	P41	46, XY[11]	P42	T.O.
FES29^a^	P37	46,XY, add(13)(p1)[1]/46,XY[30]	P37	T.O.

a Previously analyzed with karyotyping and Affymetrix SNP6.0 arrays (Narva et al., 2010).
