# Supplementary material for: High-throughput karyotyping of human pluripotent stem cells
Source: Stem Cell Res. 2012 Nov;9(3):192–5. doi: 10.1016/j.scr.2012.06.008 (PMC3502865; doi:10.1016/j.scr.2012.06.008)
Supplement: Supplementary Fig. 1 — Workflow of the Karyolite Assay. [file mmc1.pdf]

Isolation of genomic DNA (1 h)

⇓

Labelling of ≥50 ng of DNA with Biotin (45 min + 1 h incubation)

⇓

Purification of labeled DNA (35 min)

⇓

Hybridization set up (25 min)

⇓

Hybridization of Biotinylated DNA to BACs-on-Beads™ (16 h - 20 h)

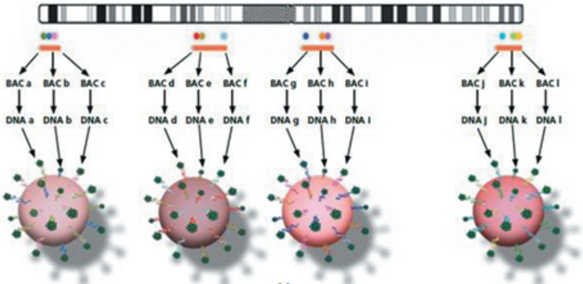

⇓

Washing and Streptavidin-PE Reporter Binding
30 min (+30 min incubation)

⇓

Measurement with Luminex® 100/200™ (1 h)

⇓

Result generation with BoBsoft™ Software (30 min)
