# Supplementary material for: High-throughput karyotyping of human pluripotent stem cells
Source: Stem Cell Res. 2012 Nov;9(3):192–5. doi: 10.1016/j.scr.2012.06.008 (PMC3502865; doi:10.1016/j.scr.2012.06.008)
Supplement: Supplementary Table 1 — Comparison of the three methods used to detect karyotypic abnormalities in this study. [file mmc2.doc]

**Supplementary Table I. Comparison of the three methods used to detect karyotypic abnormalities in this study.**

| **Method** | **G-banding** | **Affymetrix arrays** | **BoBs** |
| --- | --- | --- | --- |
| Resolution | 3-20 Mb | ≤0.7 kb | Proximal and terminal regions of chromosome arms |
| Amount of DNA | N/A | 500 ng | 50 – 240 ng |
| Sample prep time | 2 days | 1 week | 1-2 days |
| Analysis time | 2-4 weeks* | 1 week | 1-2 h |
| Population coverage | ≤50 cells | whole pool | whole pool |
| Sample price | €€€ | €€€-€€€€ | €€-€€€ |

*The time taken for G-band analysis is generally about 1.5 hrs processing and 1 hr analysis. However as mostly analysis is done in diagnostic labs the queue takes often at least 2-4 weeks for non-patient material.
