# Supplementary material for: High-throughput karyotyping of human pluripotent stem cells
Source: Stem Cell Res. 2012 Nov;9(3):192–5. doi: 10.1016/j.scr.2012.06.008 (PMC3502865; doi:10.1016/j.scr.2012.06.008)
Supplement: Supplementary file 3 [file mmc3.pdf]

COMPARISON OF 3 MOLECULAR METHODS TO DETECT ANEUPLOIDIES IN PRENATAL DIAGNOSIS

Jeremie Gras*, Nathalie Lannoy, Xavier Pepermans, Marie Ravoet, Marianne Philippe

Center for Human Genetics, UCL Saint-Luc University, Brussels (*Now at Clinique Saint-Luc Bouge)

1.Background

- In prenatal diagnostics, chromosomal abnormalities are diagnosed using FISH, array CGH and caryotyping
- These techniques are long, costly, and require a considerable amount of expertise
- The purpose of this work is to evaluate the performance of three molecular techniques (MLPA from MRC-Holland, MAQ from Multiplicon and BoBs from PerkinElmer) for the detection of chromosomal abnormalities as well as their sensitivities in the detection of mosaics

	MLPA	MAQ assay	Bacs-on-Beads
Manufacturer	MRC-Holland	Multiplicon	PerkinElmer
Aneuploidies detected	+13, +18, +21 Aneusomies X,Y	+13, +18, +21 Aneusomies X,Y	+13, +18, +21 Aneusomies X,Y
Triploid analysis	NO	Yes	Yes
Detection of micro deletions	NO	NO	Yes
Separation of amplified products	Genescan	Genescan	Luminex
CE Marking	NO	NO	Yes

2. Design

- From April to September 2010, a total of 30 AF and 17 CVS samples were prospectively collected
- These samples were firstly analyzed by FISH
- Then, DNA was purified using a homebrew phenol chloroform method, and samples were analyzed using the three molecular technologies
- Artificial mosaics were also obtained by dilutions of pathologic DNA into a negative DNA pool
  - Mosaics with 50%, 40%, 30%, 20%, 10% of abnormal cells were obtained for the following chromosomal abnormalities: +21, +18, XXY, XYY, XO, XXX

Examples of results

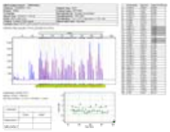

47,XY,+18 using MLPA

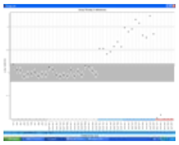

47,XX,+21 using MAQ

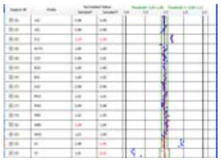

47,XX,+21 using BoBs

3. Results: comparison with FISH

	MLPA	MAQ	BoBs	FISH
AF 2	46,XY	46,XY	46,XY	Normal
AF 3	46,XX	46,XX	46,XX	Normal
AF 4	46,XX	46,XX	46,XX	Normal
AF 5	46,XY	*sample µ*	46,XY	Normal
AF 7	46,XX	46,XX	46,XX	Normal
AF 8	46,XY	46,XY	46,XY	Normal
AF 9	46,XY	46,XY	46,XY	Normal
AF 10	46,XY	46,XY	46,XY	Normal

	MLPA	MAQ	BoBs	FISH
AF 11	46,XX	*sample µ*	46,XX	Normal
AF 12	46,XX	46,XX	46,XX	Normal
AF 13	46,XY	46,XY	46,XY	Normal
AF 14	46,XY	46,XY	46,XY	Normal
AF 15	46,XY	46,XY	46,XY	Normal
AF 16	47,XXY	47,XXY	47,XXY	XXY
AF 17	46,XX	46,XX	46,XX	Normal
AF 18	Not int.	*47,XY,+21*	*47,XY,+21*	+21
AF 19	46,XX	Not int.	46,XX	Normal
AF 20	*[ADN] µ*	*[ADN] µ*	46,XY	Normal

	MLPA	MAQ	BoBs	FISH
AF 21	46,XX	46,XX	46,XX	Normal
AF 22	46,XY	46,XY	46,XY	Normal
AF 23	46,XY	46,XY	46,XY	Normal
AF 24	46,XY	Not int.	46,XY	Normal
AF 25	46,XX	*sample µ*	*sample µ*	Normal
AF 26	46,XY	46,XY	46,XY	Normal
AF 27	46,XY	46,XY	46,XY	Normal
AF 28	46,XX	Not int.	46,XX	Normal
AF 29	46,XX	46,XX	46,XX	Normal

	MLPA	MAQ	BoBs	FISH
AF 34	46,XY	46,XY	46,XY	Normal
AF 35	46,XY	46,XY	46,XY	Normal
AF 36	46,XX	46,XX	46,XX	Normal

	MLPA	MAQ	BoBs	FISH
CVS 1	*47,XX,+18*	Not. Int.	*47,XY, +18*	*47,XY, +18*
CVS 2	46,XX	Not. Int.	46,XX	Normal
CVS 3	45,XO	45,XO	45,XO	45,XO
CVS 4	*47,XX,+21*	*47,XX,+21*	*47,XX,+21*	*47,XX,+21*
CVS 5	46,XY	46,XY	46,XY	Normal
CVS 6	46,XY	46,XY	46,XY	Normal
CVS 7	46,XY	46,XY	46,XY	Normal
CVS 8	46,XY	46,XY	46,XY	Normal
CVS 9	Not. Int.	*47,XX,+21*	*47,XX,+21*	*47,XX,+21*
CVS 10	46,XX	46,XX	46,XX	Normal

	MLPA	MAQ	BoBs	FISH
CVS 11	*47,XX,+21*	*47,XX,+21*	*47,XX,+21*	*47,XX,+21*
CVS 12	*47,XY,+18*	*47,XY,+18*	*47,XY,+18*	*47,XY,+18*
CVS 13	*47,XX,+18*	*47,XX,+18*	*47,XX,+18*	*47,XY,+18*
CVS 14	46,XX	46,XX	46,XX	Normal
CVS 15	*47,XX,+21*	*47,XX,+21*	*47,XX,+21*	*47,XX,+21*
CVS 16	Not. Int.	46,XY	46,XY	Normal
CVS 17	46,XY	46,XY	46,XY	Normal

Legend:

Not. Int: result that could not be interpreted

Sample µ : insufficient sample

[DNA] µ: insufficient DNA concentration

4. Discussion

- MLPA produced 6,4 % of uninterpretable results, MAQ gave 10,6 %
- BoBs produced no uninterpretable results, its concordance with FISH was 100 %
- BoBs allowed the analysis of one sample that could not be performed using MLPA and MAQ due to insufficient DNA concentration (sample AF20)

5. Sensitivity for mosaic detection

	MLPA	MAQ	BoBs
+21 mosaic	50 % not detected	50 %	20 %
+18 mosaic	50 % not detected	50 %	30 %
XXY mosaic	40 %	30 %	30 %
XYY mosaic	30 %	30 %	30 %
XO mosaic	50 % not detected	40 %	30 %
XXX mosaic	50 % not detected	50% at the limit	30 %

% of abnormal cells detected

+21 mosaic study using BoBs

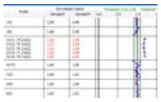

T21 – 50 %

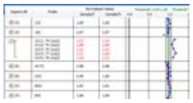

T21 – 40 %

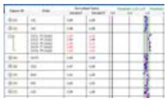

T21 – 30 %

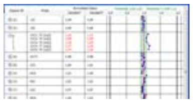

T21 – 20 %

6. Detection of micro deletion syndromes using BoBs

- BoBs is the only of the three methods to allow the detection of 9 micro deletion syndromes
- 5 frozen peripheral blood DNA samples with MDS were selected from our bio bank were selected:
  - Di George, Williams-Beurens, Prader-Willi, Angelman, Smith-Magenis
- All were successfully identified using BoBs technology

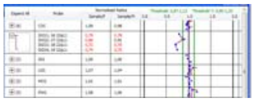

BoBs, Di George

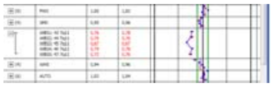

BoBs, Williams Beurens

7. Conclusions

- In this work, BoBs gave no uninterpretable results, with a total concordance with FISH; BoBs gave also the better results regarding the sensitivity to detect mosaics
- BoBs is the only of the three techniques to allow the detection of nine micro deletion syndromes; in this study, all tested micro deletions syndromes were correctly identified
- In our study, MLPA produced to many uninterpretable results; its capability to detect mosaics was insufficient
- MAQ is a fast and technique that could be particularly interesting on an operational point of view; however, too many samples are not interpretable despite numerous attempts to improve the quality of results
- Further operational studies are needed to assess the potential impact of BoBs implementation in terms of cost reduction and turn around time improvement, especially in comparison with FISH
- Notably, it could be interesting to analyze BoBs results generated with samples purified using fast DNA extraction techniques to reduce answer time

Contact: jeremie.gras@gmail.com
